# Supplementary material for: Shuttling single metal atom into and out of a metal nanoparticle
Source: Nat Commun. 2017 Oct 10;8:848. doi: 10.1038/s41467-017-00939-0 (PMC5635118; doi:10.1038/s41467-017-00939-0)
Supplement: Supplementary file 1 — Supplementary Information [file 41467_2017_939_MOESM1_ESM.pdf]

Supplementary Notes

Supplementary Note 1. Synthesis of $[Au_{24}(PPh_3)(SR)_5Cl_2]^+Cl^-$ nanocluster.

This nanocluster was prepared via three primary steps. In the first step, triphenylphosphine (PPh_3)-stabilized Au clusters were synthesized by a modified method[33]. Briefly, $HAuCl_4$ (0.16g) was dissolved in 15 mL ethanol, followed by adding 0.313g triphenylphosphine (PPh_3) into the solution under vigorous magnetic stirring at room temperature. The solution gradually turned from yellow to colorless and finally white over ~3 min. $NaBH_4$ solution (0.07g dissolved in 5 mL ethanol) was rapidly added to the reaction all at once. The reaction was allowed to proceed for ~2h at room temperature. Then, the solution was dried by rotary evaporation. In the second step, the triphenylphosphine (PPh_3)-stabilized Au clusters were dissolved in 6 mL dichloromethane, followed by addition of 0.2 mL thiol (2-Phenylethylmercaptan (PET)). The reaction was allowed to proceed for ~3h at 40°C. Finally, the obtained $[Au_{25}(PPh_3)(SR)_5Cl_2]Cl_2$ nanoclusters were washed with hexane to remove excess thiol and triphenylphosphine, followed by crystallization in CH_2Cl_2/diethyl ether system. The third step was the addition of PPh_3 to react with the obtained $[Au_{25}(PPh_3)(SR)_5Cl_2]Cl_2$ nanocluster to form $[Au_{24}(PPh_3)(SR)_5Cl_2]Cl$ nanocluster. 5mg $[Au_{25}(PPh_3)(SR)_5Cl_2]Cl_2$ nanocluster was dissolved in 2 mL CH_2Cl_2 solution, followed by adding 1g of PPh_3. The reaction was allowed to process over night at 40°C. Then, 10 mL of hexane was added to remove the excess PPh_3, followed by crystallization in DCM/pentane system.

Supplementary Note 2. Structure determination

$[Cu_1Au_{24}(PPh_3)_{10}(SR)_5Cl_2](SbF_6)_2$. At first, all the metal atoms in the cluster were solved as Au. X-ray diffraction data refinement involving partial occupancy was used to locate the Cu atom. Based on geometrical analysis, the Au atoms can be roughly categorized into 5 groups (details could be found in Supplementary Figures 1 and 2), therefore five separate free variables with initial values of 0.96 assuming equal probability for all positions were introduced to model the potential residency of Cu in these groups. After refinement and convergence, the free variables corresponding to positions P2, P4, P5 were all found to be approximately 1.03, indicating the atoms in these positions are 100% Au. The free variables corresponding to positions P1 and P3 indicated residency of Cu. Next, the atoms in position P3 were further divided into 5 different groups (Supplementary Figures 1d and 2) to better represent the cluster's symmetry; along with the two atoms in position P1, they were given 6 individual free variables to model Cu's occupancy. In the converged result, the 5 groups in (formerly) position P3 have Au occupancy of 0.94319, 0.93586, 0.92573, 0.94830, and 0.88559, respectively (see CIF file for detailed information specifying atoms and positions). The most probable positions for Cu were determined to be position P1, which has an Au occupancy of 0.85072. Regarding SbF_6 anions, the well-resolved Sb(1) was determined to be 100%; the second anion were found in different possible locations, two of which were modeled to be Sb(2) and Sb(3). A free variable was used to determine that Sb(2) has an occupancy of 0.61177. Since the F atoms that connect to Sb(3) could not be solved, other possible locations for Sb were not modeled.

$[Ag_1Au_{24}(PPh_3)_{10}(SR)_5Cl_2](SbF_6)_2$. X-ray diffraction data refinement involving partial occupancy was used to locate the Ag atom. Based on geometrical analysis, the Au atoms can be roughly categorized into 5 groups (image on the left). The refinement either becomes unstable or converge to the results in which the free variables corresponding to positions P2, P3, P4, P5 were all found to be larger than 1, indicating the atoms in these positions cannot be Ag, therefore these atoms are treated as 100% Au in subsequent refinement cycles. The free variable corresponding to position P1 indicated residency of Ag (Supplementary Figures 1d and 3). The two atoms in position A have Ag occupancy of 0.499(3) and 0.479(3), respectively.

Supplementary Figures

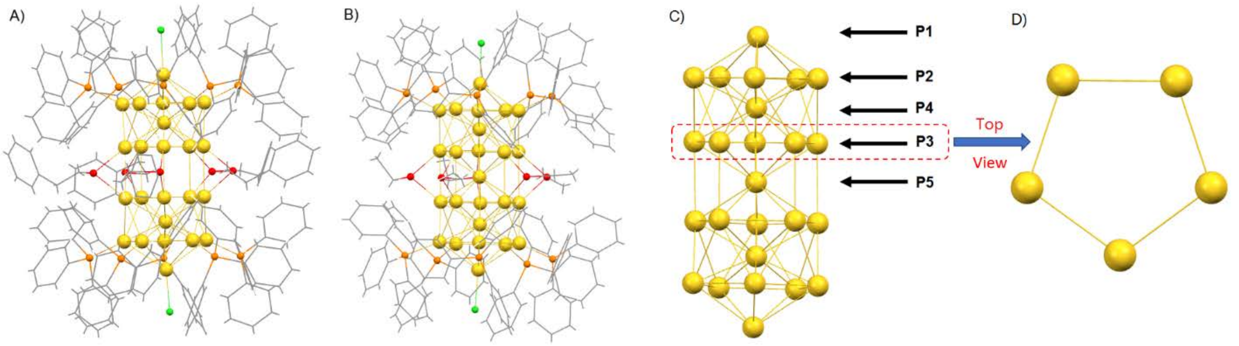

Supplementary Figure 1. The crystal structure of undoped A) Au_{24} and B) Au_{25} nanorod; C) the metal core of Au_{25} nanorod, the layer by layer gold atoms are labeled from P1 to P5; D) the P3 positions are divided into 5 different groups according to the cluster's symmetry.

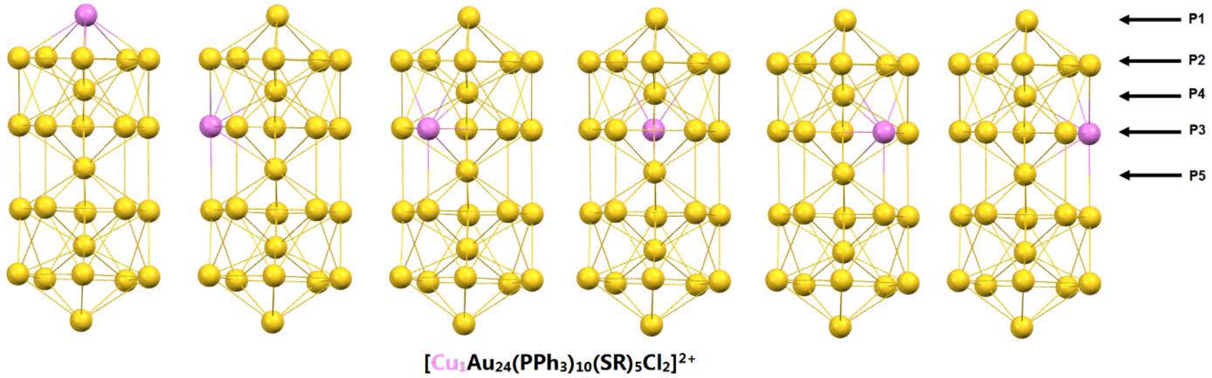

Supplementary Figure 2. The crystal structure of Cu_1Au_{24}, resolved in this work. Our analysis on Cu_1Au_{24} nanorod shows that sites P1 and P3 can be occupied by Cu. Other sites (i.e., P2, P4, and P5) are found to be occupied only by Au. Of note, site P3 can be divided into 5 different groups according to the cluster's symmetry. Only metal atoms are shown for clarity.

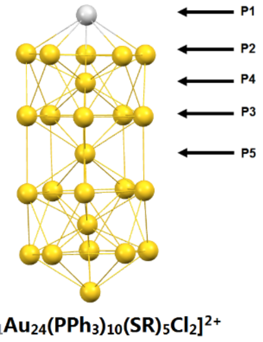

Supplementary Figure 3. The crystal structure of Ag_1Au_{24}, resolved in this work. For Ag_1Au_{24} nanocluster, our analysis shows that site P1 can be occupied by Ag. Other sites (i.e., P2, P3, P4, and P5) are found to be occupied *only* by Au. Only metal atoms are shown for clarity.

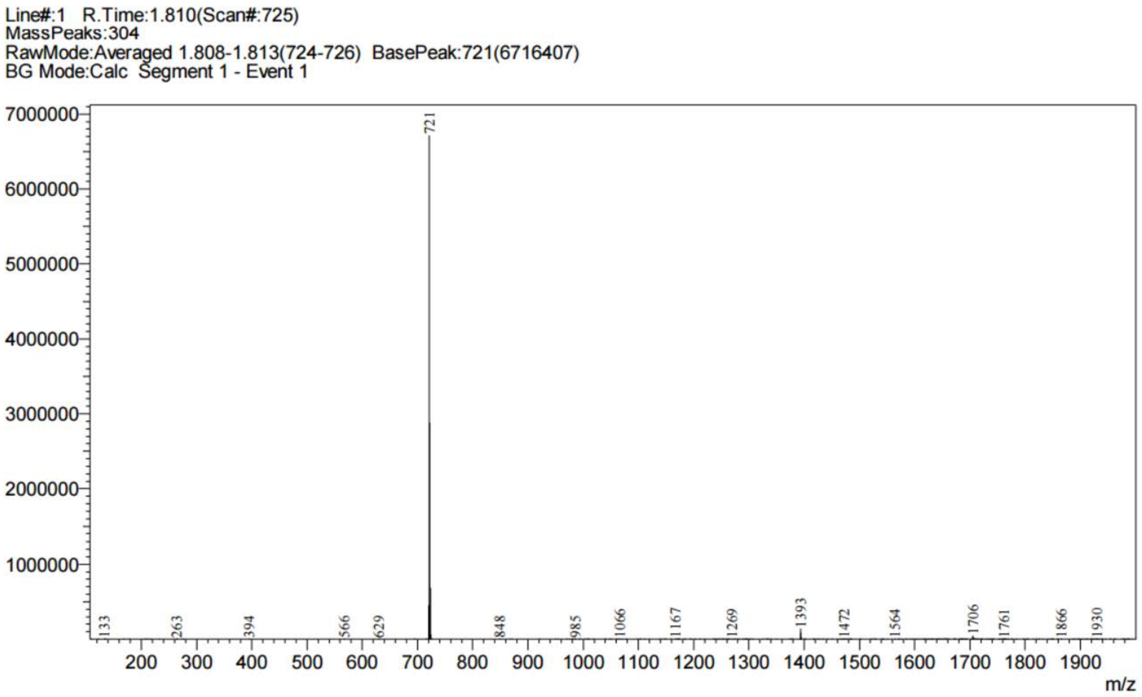

Supplementary Figure 4. The lower-mass range of the product of Au_{25} reaction with PPh_3 ligands (the single peak in the spectrum is from $Au(PPh_3)_2^+$, m/z = 721).

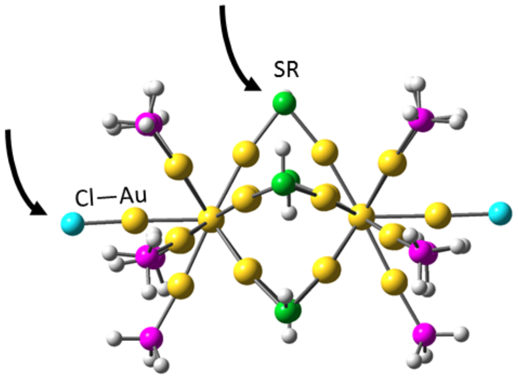

$$\Delta E(Ag^+) = E_{Ag+.....Cl—Au} - E_{Ag+\ .....SR} = -9.6\ kcal/mol$$

$$\Delta E(Cu^+) = E_{Cu+.....Cl—Au} - E_{Cu+\ .....SR} = -8.0\ kcal/mol$$

Supplementary Figure 5. DFT results of two possible sites for interaction of M^+ (M = Cu or Ag) with the capping ligands of the nanoparticle. $E_{Ag+.....Cl–Au}$ and $E_{Ag+.....Cl–Au}$ are the energies of the system when, respectively, an Ag^+ and Cu^+ is adsorbed on a Cl atom of the Au_{24} nanocluster. $E_{Ag+.....SR}$ and $E_{Ag+.....SR}$ are the energies of the system when, respectively, an Ag^+ and Cu^+ is adsorbed on a thiolate ligand of the Au_{24} nanocluster. For comparison, the relative affinity of Ag^+/Cu^+ to a free Cl^- or $–SH^-$ in the gas phase is

calculated. DFT results show that interaction energy of both Ag^+ and Cu^+ with a $–SH^-$ is about 15 kcal/mol more favorable than that with a free Cl^- ion. These results indicate, the attraction of Cl and -SR ligands with respect to Ag^+/Cu^+ differentiates if they are coordinated with the surface gold atoms. Color code: Color code: Au, yellow; H, light gray; Cl, light blue; S, green, P; magenta.

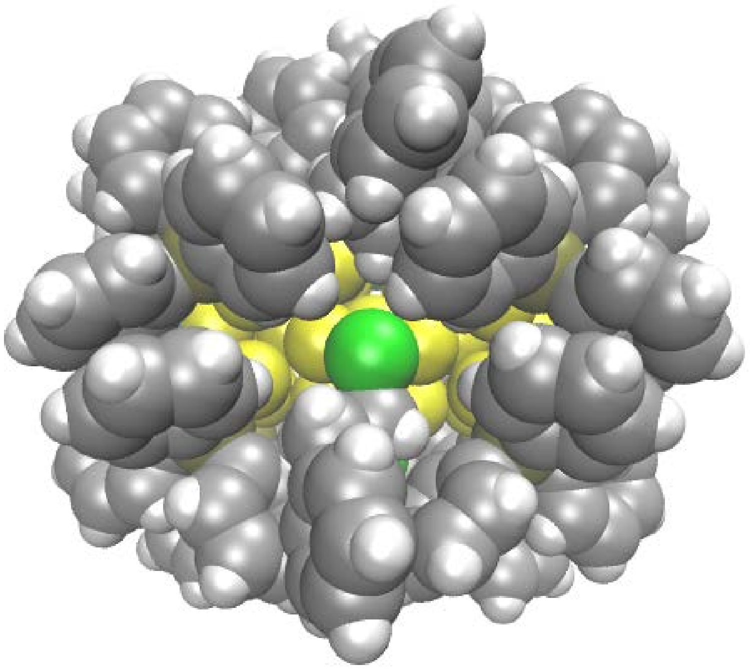

Supplementary Figure 6. Top view of the Au_{24} nanocluster presented in space-filling model. The larger vdW radius of Ag (in comparison to that of Cu) prevents silver atoms from interacting efficiently with S atom of –SR groups. Note, only one S atom is apparent from this view. Other sulfur atoms are not able to be seen from this view because of ligands. The atomic coordinates of the nanoparticle are adopted from Ref. 36. Color code: Au, yellow; H, light gray; C, gray; S, green.

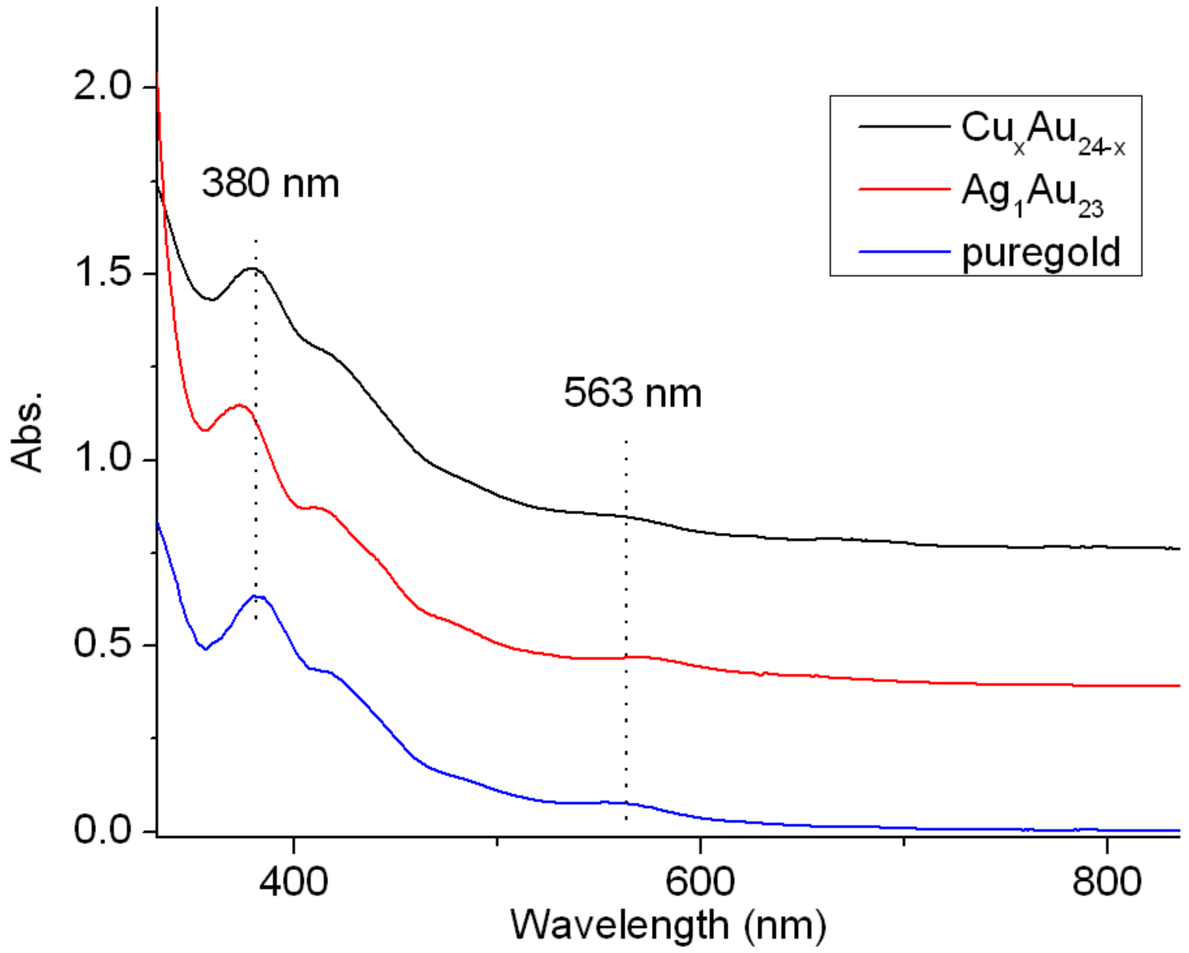

Supplementary Figure 7. The UV-Vis spectra of M_1Au_{24} (M=Au/Ag/Cu) nanoclusters after reaction with PPh_3 (reaction time: 24h).

Supplementary Tables

Supplementary Table 1. Crystal data and structure refinement for Ag_1Au_{24}.

Identification code	Ag_1Au_{24}
Empirical formula	$C_{220}H_{195}Ag_{0.98}Au_{24.02}Cl_2F_{12}P_{10}S_5Sb_2$
Formula weight	8688.00
Temperature/K	150(2)
Crystal system	monoclinic
Space group	$P2_1/n$
a/Å	20.5001(7)
b/Å	36.3894(11)
c/Å	31.7441(11)
α/°	90
β/°	91.775(2)
γ/°	90
Volume/Å^3	23669.3(14)
Z	4
ρ_{calc}g/cm^3	2.438
μ/mm^{-1}	31.288
F(000)	15731.0
Crystal size/mm^3	0.240 × 0.200 × 0.180
Radiation	CuKα (λ = 1.54178)
2Θ range for data collection/°	3.694 to 178.978
Index ranges	$-26 \leq h \leq 26$, $-42 \leq k \leq 47$, $-36 \leq l \leq 41$
Reflections collected	157767
Independent reflections	52405 [R_{int} = 0.0198, R_{sigma} = 0.0238]
Data/restraints/parameters	52405/0/2463
Goodness-of-fit on F^2	1.037
Final R indexes [I>=2σ (I)]	R_1 = 0.0313, wR_2 = 0.0818
Final R indexes [all data]	R_1 = 0.0370, wR_2 = 0.0870
Largest diff. peak/hole / e Å^{-3}	2.52/-1.76

Supplementary Table 2. Crystal data and structure refinement for Cu_1Au_{24}

Identification code	Cu_1Au_{24}
Empirical formula	$C_{220}H_{195}Au_{24}Cl_2CuF_{10}P_{10}S_5Sb_2$
Formula weight	8603.88
Temperature/K	240.0
Crystal system	monoclinic
Space group	$P_{21/n}$
a/Å	20.5150(12)
b/Å	36.596(2)
c/Å	31.8694(18)
α/°	90
β/°	91.878(2)
γ/°	90
Volume/Å3	23914(2)
Z	4
ρcalcg/cm3	2.390
μ/mm-1	30.394
F(000)	15584.0
Crystal size/mm3	0.32 × 0.14 × 0.12
Radiation	CuKα (λ = 1.54178)
2θ range for data collection/°	3.678 to 136.572
Index ranges	-24 ≤ h ≤ 23, -42 ≤ k ≤ 44, -38 ≤ l ≤ 38
Reflections collected	176725
Independent reflections	43470 [Rint = 0.0612, Rsigma = 0.0538]
Data/restraints/parameters	43470/251/2393
Goodness-of-fit on F^2	1.075
Final R indexes [I>=2σ (I)]	R_1 = 0.0645, wR_2 = 0.1666
Final R indexes [all data]	R_1 = 0.0742, wR_2 = 0.1768
Largest diff. peak/hole / e $Å^{-3}$	6.55/-8.39

Supplementary Table 3. Energy (kcal mol^{-1}) of the optimized Ag_1Au_{24} nanoclusters with Ag located at the different positions (see Supplementary Figure 3). The energy of the most stable system is set to 0.00. Energies of the other systems are reported relative to the most stable system. A more positive energy for a position indicates that such a site is energetically less probable to be occupied by an Ag. Results of DFT with van der Waals (vdW) corrections using the Grimme-D2 and the Exchange-hole dipole-moment (XDM) methods are also presented.

Position	DFT	DFT-D2	DFT-XDM
P1	5.69	2.89	6.12
P2	9.09	7.91	9.62
P3	3.97	4.09	3.87
P4	20.12	16.83	21.24
P5	0.00	0.00	0.00

Supplementary Table 4. Energy (kcal mol^{-1}) of optimized Cu_1Au_{24} nanoclusters with Cu located at the different positions (see Supplementary Figure 2). The energy of the most stable system is set to 0.00. Energies of the other systems are reported relative to the most stable system. A more positive energy for a position indicates that such a site is energetically less probable to be occupied by a Cu. Results of DFT with van der Waals (vdW) corrections using the Grimme-D2 and the Exchange-hole dipole-moment (XDM) methods are also presented.

Position	DFT	DFT-D2	DFT-XDM
P1	0.00	2.44	0.00
P2	6.53	7.64	6.77
P3	2.29	6.42	2.04
P4	10.22	8.42	9.28
P5	0.22	0.00	0.13
