# Supplementary material for: Shuttling single metal atom into and out of a metal nanoparticle
Source: Nat Commun. 2017 Oct 10;8:848. doi: 10.1038/s41467-017-00939-0 (PMC5635118; doi:10.1038/s41467-017-00939-0)
Supplement: Supplementary file 3 — Description of Additional Supplementary Files [file 41467_2017_939_MOESM3_ESM.pdf]

Description of Additional Supplementary Files

File Name: Supplementary Movie 1
Description: Migration of a PH_3 ligand to form a bond with a gold atom at waist position with simultaneous breaking of an Au—S bond (Figure 4, Reac → Int1). Color code: Au, yellow; Cl, cyan; S, green; P, pink; H, white.

File Name: Supplementary Movie 2
Description: A gold atom is completely pulled up to the surface of the nanoparticle (Figure 4, Int1 → Int2) with the help of coordinating PH_3 ligand. Such an Au atom is thus exposed to excess PH3 ligands in the reaction medium to form $Au(PH_3)2$ on the surface of the nanoparticle (Figure 4, Int2 → Int3). Color code: Au, yellow; Cl, cyan; S, green; P, pink; H, white.

File Name: Supplementary Movie 3
Description: Possible pathway for an Ag to locate at the apical site by squeezing a gold atom at the site of icosahedral center (Figure 5c, Path1). Color code: Ag, blue; Au, yellow; Cl, cyan; S, green; P, pink; H, white.

File Name: Supplementary Movie 4
Description: Possible pathway for an Ag to locate at the apical site by squeezing a gold atom at the site of waist (Figure 5c, Path 2). Color code: Ag, blue; Au, yellow; Cl, cyan; S, green; P, pink; H, white.
